# Bioimplant-on-a-Chip for Facile Investigation of Periodontal Ligament Formation on Biogenic Hydroxyapatite/Ti_6_Al_4_ V Implants

**DOI:** 10.1021/acsami.5c04687

**Published:** 2025-05-13

**Authors:** Sangbae Park, Jae Eun Kim, Juo Lee, Woochan Kim, Woobin Choi, Myung Chul Lee, Jae Woon Lim, Kyoung-Je Jang, Hoon Seonwoo, Jangho Kim, Jong Hoon Chung

**Affiliations:** † Department of Biosystems Engineering, 26725 Seoul National University, Seoul 08826, Korea; ‡ Research Institute of Agriculture and Life Sciences, Seoul National University, Seoul 08826, Republic of Korea; § Integrated Major in Global Smart Farm, College of Agriculture and Life Sciences, Seoul National University, Seoul 08826, Republic of Korea; ∥ Department of Convergent Biosystems Engineering, College of Life Science and Natural Resources, 65380 Sunchon National University, Suncheon 57922, Republic of Korea; ⊥ Department of Convergence Biosystems Engineering, 34931 Chonnam National University, Gwangju 61186, Republic of Korea; # Department of Rural and Biosystems Engineering, Chonnam National University, Gwangju 61186, Republic of Korea; ¶ Interdisciplinary Program in IT-Bio Convergence System, Chonnam National University, Gwangju 61186, Republic of Korea; ∇ Medicinal Materials Research Center, Biomedical Research Division, Korea Institute of Science and Technology (KIST), Seoul 02792, Republic of Korea; ○ Department of Bio-Systems Engineering, Institute of Smart Farm, 26720 Gyeongsang National University, Jinju 52828, Republic of Korea; ⧫ Institute of Agriculture & Life Science, Gyeongsang National University, Jinju 52828, Republic of Korea; †† Interdisciplinary Program in IT-Bio Convergence System, Sunchon National University, Suncheon 57922, Republic of Korea; △ Institute of Nano-Stem Cells Therapeutics, NANOBIOSYSTEM Co., Ltd, Gwangju 61008, Republic of Korea; ▼ ELBIO Inc, Seoul 08812, Republic of Korea

**Keywords:** bioimplant-on-a-chip, bioimplants, periodontal ligament, in vitro model, tissue engineering

## Abstract

Highly osseointegrative dental implants surrounded by reconstructed periodontal tissues represent a promising strategy for functional tooth replacement, as they mimic the structural and physiological characteristics of natural teeth. However, there is currently a lack of in vitro platforms that can effectively evaluate the integration of engineered periodontal ligament (PDL) tissues with bioimplants. In this study, we developed a bioimplant-on-a-chip (BoC) platform designed to recapitulate the native PDL-cementum interface and assess the early stage biological performance of bioimplants in vitro. The BoC consists of a dental implant, a calcium phosphate cement (CPC) insert, a nanopatterned polydimethylsiloxane (PDMS) substrate, and PDL-like tissue derived from human dental pulp stem cells (DPSCs). To establish viable culture conditions within the platform, surface coatings and cell seeding densities were optimized to support the formation of PDL-like tissue. Nanogrooved substrates were incorporated to guide cellular alignment, which was assessed through orientation analysis. Collagen fiber organization and matrix deposition were further examined as indicators of ligamentous tissue maturation. Cementogenic activity was evaluated by immunofluorescent staining of cementum protein-1 (CEMP-1) in response to varying biogenic hydroxyapatite (bHA) contents in the bioimplants. The results demonstrated successful reproduction of a PDL-like tissue interface and material-dependent differences in CEMP-1 expression. This platform provides a modular and reproducible tool for the comparative evaluation of bioimplants in a physiologically relevant setting and may be useful in advancing regenerative strategies in dental implantology.

## Graphical abstract

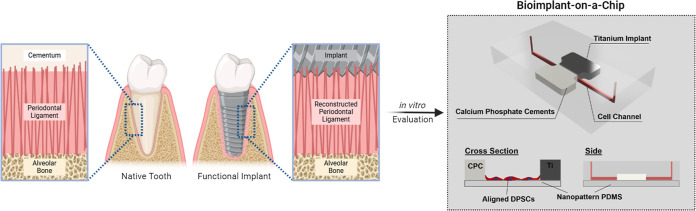

## 1 Introduction

Organ-on-chips have been introduced to replace conventional methods of evaluation such as in vivo and simple in vitro models that may not be reproducible, time-efficient, or relevant in humans.^1^ Organ-on-chips, on the other hand, are able to provide platforms that take into account the internal structures and various physiochemical factors of tissues/organs, allowing for the replication of the complex physiological microenvironments found in situ. In particular, PDMS-based platforms offer excellent optical transparency, which facilitates dynamic, noninvasive, real-time monitoring of cellular behaviors and implant–tissue interactions, thereby enhancing their utility for biological modeling and analysis.^2,3^ The goal of these systems is to create a biomimetic model that recapitulates the smallest functional unit on an organ/tissue,^4^ ultimately allowing for easy experimental control over the multifactorial issues that often arise in complicated in vivo studies.^5^ Organ-on-chip models have been widely applied to mimic tissues such as the liver, kidney, lung, heart, and skin,^6–12^ but few studies have addressed dental tissues. The periodontium is a specialized tissue that surrounds the tooth and consist of cementum, periodontal ligament (PDL), alveolar bone, and gingival tissue. Among these, the PDL is a highly aligned connective tissue that anchors the tooth to the alveolar bone and plays a central role in shock absorption and protection against infections and/or inflammatory injuries.^13–15^ Its highly organized structure is difficult to reproduce in vitro, yet is likely essential for restoring functional periodontal architecture.^16^ Accurately reproduced periodontal models would enable more detailed studies of periodontal tissues and facilitate the testing of dental materials, disease models, and regenerative therapies. However, current in vitro platforms lack the complexity needed to mimic the native periodontal microenvironment.^17^ Therefore, organ-on-a-chip platforms that are able to effectively recapitulate dental tissues, especially the PDL, should be developed.

An organ-on-a-chip platform able to mimic dental tissues can be preferentially applied to the development of dental implants. A dental implant is an artificial tooth root that is surgically inserted into the alveolar bone, serving as a foundation for a dental prosthesis such as a crown or bridge,^18,19^ typically achieving direct osseointegration without the presence of a PDL.^20–22^ This structural difference limits the implant’s ability to perform PDL-specific functions, such as reducing excessive occlusal load and perceiving noxious stimuli, potentially leading to complications like peri-implantitis and periodontal collapse in the long term.^23,24^ Although a highly osseointegrative dental implant surrounded by a reconstructed cementum-PDL complex is considered to be the ideal tooth replacement option, reconstructing the periodontium around dental implants remains a major challenge for researchers.^25,26^ Recent studies have attempted to regenerate PDLs by applying using single or multilayered cell sheets to implant surfaces prior to implantation,^21,27^ which have shown promise in promoting aligned tissue formation and cementum-like matrix formation.^28,29^ While cell sheet-based approaches show promise for PDL regeneration, the development and subsequent incorporation of bioimplants—dental implants incorporating surface modifications or bioactive materials—is a necessary complement to further enhance cementum-like tissue regeneration and facilitate comprehensive periodontium reconstruction. Conventional dental implants are fabricated using titanium and titanium alloys due to their excellent mechanical properties and biocompatibility.^30,31^ However, their bioinert properties have sometimes led to limited regenerative outcomes. Recently, hydroxyapatite (HA), particularly biogenic HA (bHA) derived from animal bone, has been shown to exhibit excellent biocompatibility and facilitate cell proliferation.^32–36^ The integration of bHA into titanium implants, thereby forming bioimplants, improves their bioactivity and has been shown to enhance cementogenic differentiation and PDL formation, demonstrating their potential in periodontal tissue regeneration and cementum-like tissue reconstruction.^37–41^ However, most studies evaluating PDL regeneration around implants rely on in vivo models, which are limited by translational significance, ethical constraints, and lack of experimental control.^42,43^ Therefore, a novel in vitro organ-on-a-chip platform is required to effectively evaluate the interaction between implants and the surrounding periodontium.^44^

To mimic the complex physiological conditions of the periodontium, an effective organ-on-a-chip platform must be able to recapitulate both the biochemical and mechanical cues of the native extracellular matrix (ECM). Achieving this requires meticulous manipulation of the platform at the micro- to nanoscale. Extensive research has been conducted on the effects micro-, nanotopography, along with surface chemistry, have on cell behavior. Although the exact mechanisms by which cells recognize surface features have yet to be fully understood, several key molecules involved in this process have been identified.^45^ Among them, integrins—heterodimeric transmembrane proteins consisting of α and β subunits—are the most prominent.^46,47^ Integrin-mediated cell adhesion is regulated by both the biochemical composition of the substrate as well as the mechanical cues induced by substrate topography. Upon contact with a surface, integrins will cluster and subsequently recruit other cytoplasmic proteins to form integrin adhesion complexes (IACs), initiating cell adhesion.^48^ The recruitment of specific integrins depends largely on the availability of integrin-binding peptides found in ECM proteins such as fibronectin, gelatin, and laminin.^48^ Integrins will initiate the assembly of IACs only upon recognizing and subsequently binding to integrin-binding peptides.^49^ Evidently, adequate biochemical stimulation through the presentation of integrin binding motifs is a vital precedent to effective integrin-mediated cell adhesion, and thus, an organ-on-a-chip platform must be engineered in a way to ensure that the appropriate molecular cues are present and abundant, as would be found in the native ECM. In addition to biochemical signaling, the highly aligned nature of the native PDL tissue must be recreated in order to effectively emulate the periodontium. This can be achieved through nanoscale surface patterning, as integrin clustering and IAC formation is heavily regulated by various mechanical stimuli.^49^ The application of external forces leads to the mechanical unfolding of talin, an intracellular protein that links integrins to the actin cytoskeleton, exposing cryptic binding sites for vinculin and other IAC components.^48^ This cascade promotes further talin unfolding and thereby stunting retrograde actin flow, reinforcing adhesion.^48^ On the cellular level, filopodia are used to probe the substrate, anchoring to regions where large, clustered IACS, otherwise known as focal adhesions, can form. Because filopodial activity is isotropic and prone to retraction via retrograde actin flow unless stabilized by adhesion complexes, cells tend to migrate toward regions that facilitate strong anchorage. Exploiting this behavior through nanoscale surface patterning enables directional cell migration and alignment—critical steps in engineering PDL-like tissue.

In this study, we developed a novel bioimplant-on-a-chip (BoC) platform that mimics the physiological structure of the periodontium to evaluate the potential of bioimplants for cementum-like tissue regeneration (Figure 1). The platform was designed to reconstruct the implant–PDL–cementum interface by integrating bioimplants, calcium phosphate cements (CPCs), and PDL-like tissues. To emulate the native architecture of the PDL, nanotopographic and biochemical cues were incorporated to induce cellular alignment and promote tissue organization. The formation of PDL-like tissue and its interaction with bioimplants were systematically investigated to determine the platform’s suitability for modeling soft tissue integration under physiologically relevant conditions.

**Figure 1:**
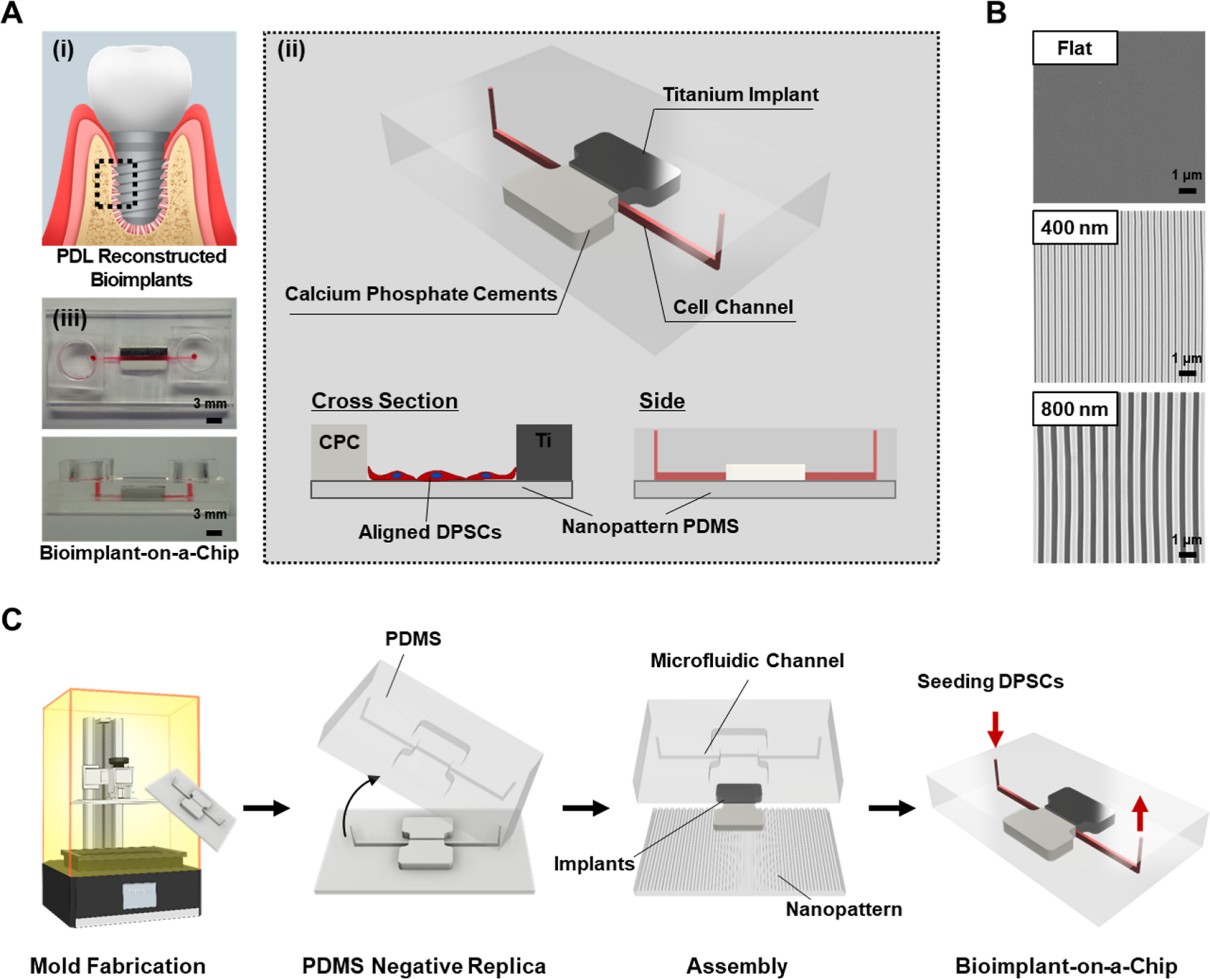
Development of BoC. (A) Considerations for designing a BoC. (i) The concept of ideal implantation, where periodontal ligaments were reconstructed surrounding bioimplants. (ii) 3D-modeled image of BoC. Microchannels for cell culture traverse between two inserts which are titanium implants and CPCs. The seeded DPSCs can interact with both inserting materials. (iii) Images of fabricated BoC. (B) FE-SEM images of the nanopatterns. Nanopatterns are fabricated using conventional soft lithography and replicated to PDMS. Flat, 400 nm, and 800 nm patterns were assembled at the bottom layer of the BoC. Nanopatterns with 400 and 800 nm grooves were used. (C) Schematic illustration of fabrication steps for BoC. The mold of BoC was fabricated via a DLP-based 3D printing, followed by PDMS replication. The PDMS replica, titanium implant, CPCs, and nanopatterns were then assembled to form a device. DPSCs were subsequently seeded in the microfluidic channel and cultured for 7 days. BoC, bioimplant-on-a-chip; CPC, calcium phosphate cement; DPSCs, dental pulp stem cells; FE-SEM, field emission-scanning electron microscopy; PDMS, polydimethylsiloxane; DLP, digital light processing.

## 2 Experimental Section

### 2.1 Design and Fabrication of Microchannel Devices

The microchannel devices were fabricated using a negative replica molding method. The positive mold of the microchannel devices was designed using computer-aided design (CAD) software (Fusion 360; Autodesk Inc., San Rafael, CA, USA). The microchannel devices had three channels identical to the microchannels in the BoC platform in a single device. These devices allow for easy optimization of culture conditions. The positive mold was fabricated with a digital light processing (DLP)-based 3D printer (Photon Mono X; Anycubic, China) using a photocurable resin (Formlabs, Somerville, MA, USA). The printed positive molds were postprocessed by washing with isopropyl alcohol (Duksan, Korea) followed by ultraviolet (UV) curing for 15 min. The polydimethylsiloxane (PDMS) (Sylgard 184; Dow Chemical, Midland, Michigan, USA) was prepared by mixing the prepolymer and cross-linker in a ratio of 10:1. The PDMS mixture was poured into a mold and baked at 60 °C for 2 h. The negative PDMS replica was then peeled off for further fabrication. Prior to assembling the PDMS replica with slide glass and flat PDMS, the surface of each component was treated with O_2_ plasma (Femto Science, Korea) for 90 s. The components were then bonded together to form a device and baked at 45 °C for 2 h to achieve irreversible bonding.

### 2.2 Investigation of Microchannel Cell Culture Conditions

Dental pulp stem cells (DPSCs) were obtained from the tooth of a patient at the Dental Hospital of Seoul National University (IRB no.: CRI05004). The DPSCs were cultured in proliferation medium, which is alpha minimum essential medium (α-MEM; Welgene, Gyeongsan, Korea) supplemented with 10% fetal bovine serum (FBS; Welgene, Gyeongsan, Korea) and 1% antibiotic/antimycotic solution (Welgene, Gyeongsan, Korea) at 37 °C in a humidified 5% CO_2_ incubator. The culture medium was changed every 2 days prior to use. Microchannel devices were used to optimize cell culture conditions. To determine the seeding concentration in the microchannels, DPSCs at different concentrations of 2.0 × 10^6^, 1.0 × 10^6^, 0.5 × 10^6^, and 0.25 × 10^6^ cells/mL were seeded into the microchannels. The microchannels were observed under a light microscope (Olympus, Tokyo, Japan), and the initial number of seeded cells per area was analyzed using ImageJ software (NIH, Bethesda, MD, USA). After 24 h, the attached DPSCs were evaluated using a live/dead assay kit (Invitrogen, Waltham, MA, USA). The live/dead assay was performed according to the manufacturer’s instructions. After removal of the culture medium, the microchannels were washed with Dulbecco’s phosphate-buffered saline (DPBS; Welgene, Gyeongsan, Korea) and incubated with a DPBS solution containing 0.5 μL/mL calcein-AM and 2 μL/mL ethidium homodimer for 30 min at 37 °C. Immunofluorescence images were captured with a fluorescence microscope (Nikon, Tokyo, Japan). The number of adherent cells and cell viability were analyzed using ImageJ software.

### 2.3 Investigation of Surface Coating Conditions for Microchannels

To investigate the ECM layers suitable for our model, gelatin (Sigma-Aldrich, St. Louis, MO, USA), poly l-lysine (Sigma-Aldrich, St. Louis, MO, USA), and fibronectin (Sigma-Aldrich, St. Louis, MO, USA) solutions were prepared at concentrations of 0.2% w/v, 100 μg mL^–1^, and 200 μg mL^–1^, respectively. The microchannels were then filled with different ECM solutions and incubated at room temperature for 24 h. DPSCs were seeded into the microchannels at a concentration of 1 × 10^6^ cells/mL. After 24 h, the adherent DPSCs were qualitatively evaluated using a live/dead assay. In addition, a WST-1 assay (Daeillab, Seoul, Korea) was performed to quantitatively compare the different ECM coatings. Briefly, DPSCs were treated with culture medium containing 10% WST-1 agent and incubated at 37 °C. After 1 h, the medium was transferred to a 96-well plate. The optical density of the samples was measured at a wavelength of 450 nm using a microplate reader (Tecan, Mannedorf, Switzerland).

### 2.4 Inducing Cell Alignment Using Nanopatterns

Nanopatterned substrates with 400 and 800 nm grooves were employed to induce the alignment of DPSCs. The nanopatterned substrates were fabricated using the soft lithography method described in our previous study.^50^ Briefly, UV-curable polyurethane acrylate (PUA) precursor solution and photoinitiator were mixed and dropped on silicon master patterns with 400 and 800 nm grooves prepared by conventional photolithography. A polyethylene terephthalate (PET) film was then attached to the PUA mixture and UV cured for 30 s. The cured PUA replica was peeled off the silicon master and further cured for 12 h to complete the reaction. The PDMS mixture was poured onto a nanopatterned positive PUA replica and baked at 60 °C for 2 h. The negative PDMS replica was then peeled off. The nanopattern on the PDMS replica was confirmed by field emission scanning electron microscopy (FE-SEM). The samples were sputter-coated with platinum to a thickness of approximately 20 nm. The samples were observed under the FE-SEM (Carl Zeiss, SUPRA 55VP, Germany) under operating conditions with an accelerating voltage of 2 kV and a working distance of 3.5–4.0 mm. To investigate alignment of the DPSCs induced by nanotopographical cues, microchannel devices with different bottom layers (flat, 400 and 800 nm) were fabricated. DPSCs were seeded into the microchannel devices at concentrations of 1.0 × 10^6^, 0.5 × 10^6^, and 0.25 × 10^6^ cells/mL. The culture medium was changed daily. On days 1, 4, and 7, a live/dead assay was performed and fluorescence images were taken. The cell orientation angle was measured using ImageJ software. Cell alignment was further evaluated using the resultant vector lengths from the images. A resultant vector length value closer to 1 indicates that the cells are aligned in one direction, while a value closer to 0 indicates that the cells are dispersed in multiple directions.^50–52^

$$Resultant\;vector\;length=\sqrt{{\left(\frac{1}{N}\sum_{i=1}^{N}\cos{\;\theta }_{i}\right)}^{2}+{\left(\frac{1}{N}\sum_{i=1}^{N}\sin\theta_{i}\right)}^{2}}$$

Picro-Sirius Red staining (ScyTek, West Logan, UT, USA) was performed to confirm the formation of collagen matrix on the nanopatterned substrates. Collagen formation in DPSCs was compared with that in periodontal ligament stem cells (PDLSCs). DPSCs and PDLSCs were seeded into the microchannel devices at a concentration of 1 × 10^6^ cells/mL in the proliferation media. The medium in the reservoirs was changed daily. After 7 days of culture, the cells were fixed with 4% paraformaldehyde solution for 30 min. The samples were treated with Picro-Sirius solution and incubated for 60 min. The samples were then rinsed twice with 0.5% acetic acid solution and washed with absolute alcohol. Microscopic images of the stained samples were obtained using a bright-field microscope (Nikon, Tokyo, Japan).

### 2.5 Development of BoC

The BoC consists of two reservoirs at the top, microchannels running between two inserts, bioimplants, CPC and nanopatterned PDMS at the bottom layer. The reservoirs were designed to be 8 mm in diameter and were placed at the top of the BoC to hold the culture medium. The middle layer consisted of microchannels for cell culture and spaces for the insets. The middle layer was fabricated by negative replica molding, and the fabrication procedure was identical to that of the microchannel devices. The width of the microchannels was chosen to be 400 μm, which mimics the thickness of the human PDL.^53^ The bioimplant and the CPC were selected for the insets included in the BoC. The bioimplants were fabricated by selective laser melting (SLM). Briefly, bHA powders derived from equine bone were prepared according to the procedure described in our previous study.^54^ The bHA powders were mixed with Ti powders (Toho Technical Service Co., Kanagawa, Japan) with volume fractions of 0.05% and 0.5%. Ti/bHA composite powders were ball milled in a planetary mill at 200 rpm for 12 h. The Ti/bHA composite powders were then transferred to an SLM printer (CSCAM, Korea) to fabricate Ti/bHA implants with shapes that fit the insertion site of the BoC. A conventional CPC was used in the alveolar bone region. The CPC paste was prepared by mixing 60% α-tricalcium phosphate (α-TCP; Ca_3_(PO_4_)_2_), 26% anhydrous dicalcium phosphate (DCPA; CaHPO_4_), 10% calcium carbonate (CaCO_3_), and 4% HA with 4% disodium phosphate (Na_2_HPO_4_) solutions. The CPC paste was filled into a mold and incubated in a humidified CO_2_ incubator at 37 °C for 3 days.^55^ Each component was treated with O_2_ plasma and assembled to manipulate a BoC device. Finally, DPSCs were seeded into the microchannel of the BoC at a concentration of 1 × 10^6^ cells/mL. The culture medium was changed daily until day 7. The fully assembled BoC device cultured with DPSCs mimics the structure of native periodontal tissue, which consists of cementum, PDL, and alveolar bone.

### 2.6 Evaluating Periodontal Ligament Regeneration on Bioimplants Using BoC

Immunocytochemistry (ICC) was performed to evaluate the osseointegration capacity of the induced PDL-like tissues. DPSCs were seeded into the BoC at a concentration of 1 × 10^6^ cells/mL in proliferation medium. The medium in the reservoirs was changed daily. After 4 days of culture, DPSCs were fixed with 4% paraformaldehyde solution for 30 min and permeabilized with 0.2% Triton X-100 for 15 min. The samples were then stained with tetramethylrhodamine (TRITC)-conjugated phalloidin for 1 h, anti-CEMP-1 primary antibody (Abcam, Cambridge, UK) for 1 h and 4′,6-diamidino-2-phenylindole (DAPI) for 10 min, followed by treatment with fluorescein isothiocyanate (FITC)-conjugated secondary antibody (Millipore, Billerica, MA, USA) for 1 h. Immunofluorescence images were captured with a fluorescence microscope (Nikon, Japan).

### 2.7 Statistical Analysis

All quantitative data are presented as mean ± standard error of the mean (SE). Student’s *t*-test was performed to analyze differences between two groups, and the significance level was set at *p < 0.05 and **p < 0.05. Analysis of variance was performed to analyze the differences between multiple groups, followed by Duncan’s multiple range test for post hoc analysis. The significance level was set at *p* < 0.05.

## 3 Results

### 3.1 Microchannel Cell Culture Conditioning

As shown in Figure 1A, the BoC are composed of the structure of the bioimplant-PDL-cementum interface. Nanopatterned PDMS was incorporated into the bottom layer of the BoC to induce the alignment of human dental stem cells for the induction of PDL-like tissues (Figure 1B). The schematic diagram in Figure 1C describes the fabrication process of the BoC. Briefly, the replica mold was fabricated by a DLP-based 3D printer using photocurable resin. From the negative replica, negative PDMS replicas were fabricated and assembled with Ti_6_Al_4_ V­(Ti)/bHA bioimplant, CPC, and nanopatterned PDMS. Dental stem cells were then seeded into the microchannel in the assembled device to complete the BoC constitution.

Prior to the investigation using the BoC, microchannel devices without inserts were fabricated to condition the microchannel fit to cell culture conditions. Since the bottom layer of this platform is nanopatterned PDMS, cell adhesion to PDMS was compared with that of glass. DPSCs were used in our platform because of their excellent proliferation and differentiation ability for dental regeneration compared to that of other stem cells.^56^ First, DPSCs were seeded into the microchannel devices with glass and PDMS bottoms, respectively. As shown in Figure S1A, DPSCs were seeded at three concentrations: 2 × 10^6^, 1 × 10^6^, and 0.5 × 10^6^ cells/mL into each device. The seeding density of DPSCs in the microchannels was calculated using ImageJ software, and it was confirmed that there was no significant difference between the glass-bottomed and PDMS-bottomed devices (Figure S1B). The seeded DPSCs were incubated for 24 h. Live/dead assay was performed to clearly observe the adherent cells in the microchannel devices (Figures S2 and 2A). The density of adherent cells and cell viability were calculated by analyzing the fluorescence images using ImageJ software. Results show that there were no significant differences in cell density and cell viability between the glass-bottomed device and the PDMS-bottomed device (Figure 2B,C). Although there were no significant differences, the adhesion behavior was different in the fluorescence images. The DPSCs adhered and spread properly on almost the entire surface of the glass-bottomed microchannel devices, whereas the DPSCs on the PDMS were aggregated or detached.

**Figure 2:**
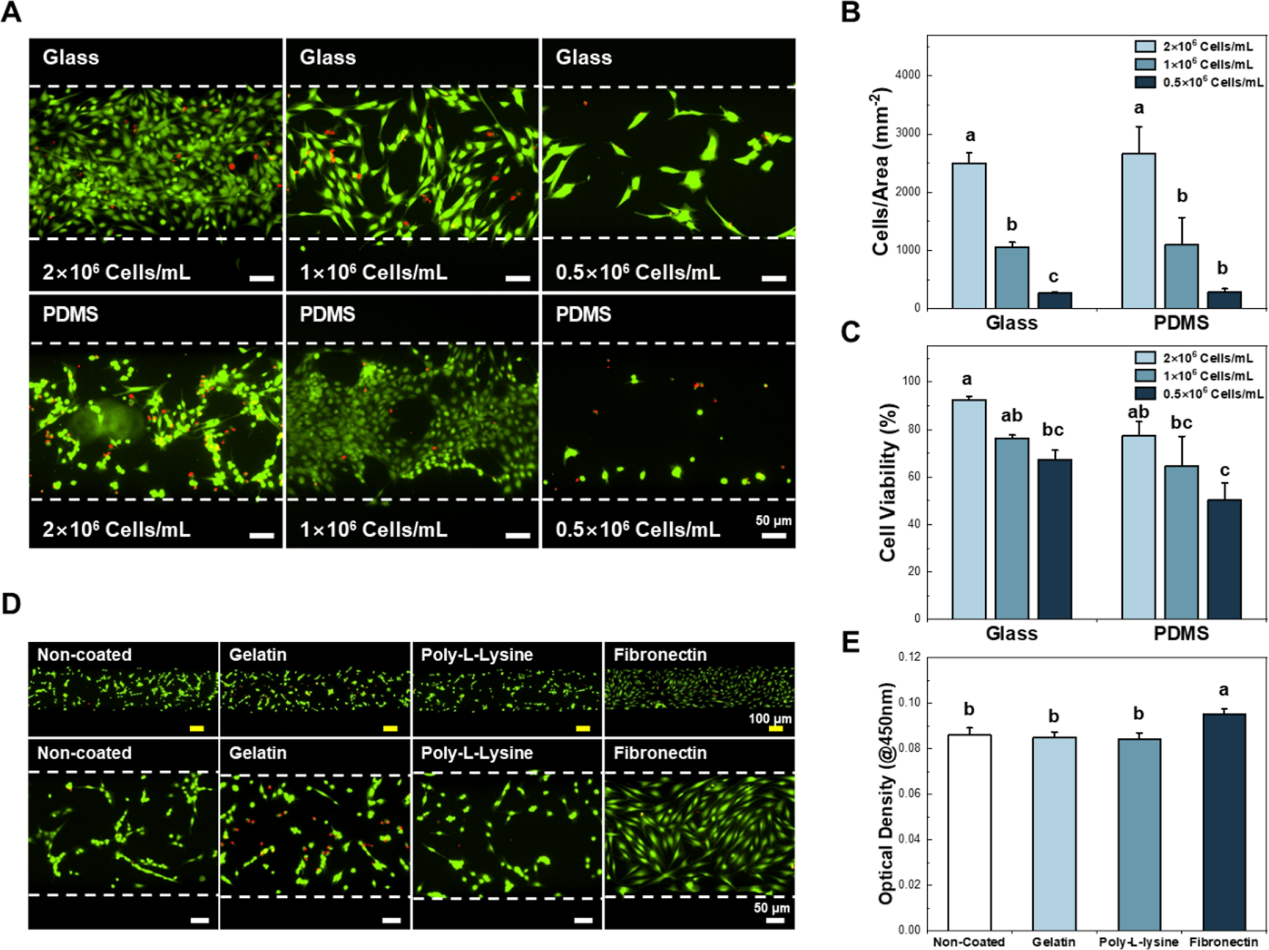
Optimization of microchannel cell culture. (A) Attachment of DPSCs on the glass and PDMS substrata. Live/dead images of DPSCs seeded onto glass- and PDMS-bottomed microchannels (magnification: 100×). (B) Density of attached DPSCs in the microchannels. (C) Cell viability of DPSCs seeded in the microchannels. There were no significant differences in cell density and cell viability between the glass-bottomed device and the PDMS-bottomed device, however their adhesion behavior differed as shown in fluorescent images. (D) Optimization of surface coating for microchannels. Gelatin, poly l-lysine, and fibronectin are coated on the PDMS to promote attachment of DPSCs. Fibronectin coating exhibited enhanced cell adhesion compared to other groups. (E) Cell viability of DPSCs on various surface coated substrata after 24 h. Fibronectin coating showed significantly enhanced cell adhesion compared to other groups. (ANOVA, Duncan’s multiple range test, *p* < 0.05). Error bars in (B,C,E) mean standard errors. ANOVA, analysis of variance.

### 3.2 Conditioning of the Surface Coating for Microchannels

Since the DPSCs on the PDMS substrate showed poor cell attachment, we coated the surface with various ECM components to promote cell adhesion. Gelatin, poly l-lysine, and fibronectin were tested as surface coating materials. Figure 2D shows the fluorescence images of the DPSCs attached to the ECM-coated surfaces. As a result, fibronectin showed improved cell adhesion compared to the other groups, with an even distribution of cells throughout the substrate. Cell attachment was further evaluated using a WST-1 assay. The fibronectin coating showed significantly improved cell adhesion compared to that of other groups, consistent with the fluorescence images (Figure 2E). In addition, the effect of seeding concentration, incubation time, and temperature on the fibronectin coating was investigated. The optimal conditions for fibronectin coating were overnight incubation at room temperature (RT) (Figure S3).

### 3.3 Cell Alignment Using Nanopatterns

After studying surface coating conditions and microchannel cell culture conditions, the influence of nanopatterns on DPSC alignment was investigated. Microchannel devices with flat bottom layers, 400 and 800 nm nanogrooved bottom layers were fabricated. The DPSCs were seeded into the microchannel devices at concentrations of 1.0 × 10^6^, 0.5 × 10^6^, and 0.25 × 10^6^ cells/mL. Static culture conditions were maintained to mitigate shear stress potentially introduced by flow perpendicular to the nanogroove orientation, thereby preserving topography-guided cellular alignment. On day 1, the DPSCs at the concentration of 0.5 × 10^6^ and 0.25 × 10^6^ cells/mL did not show any particular alignment and were distributed in different directions (Figure 3A,B). However, at a concentration of 1.0 × 10^6^ cells/mL, the DPSCs were aligned along the direction of the nanopatterns, which was perpendicular to the direction of flow. On day 4, the DPSCs in the 400 and 800 nm groups were aligned in the direction of the nanopatterns, whereas the cells in the flat substrate were aligned in the direction of flow (Figure S4). This tendency was maximized at day 7. Regardless of the type of nanopattern, more than 50% of the cells were oriented in the vertical direction. In particular, DPSCs seeded on the 800 nm patterned surface at a concentration of 1.0 × 10^6^ cells/mL concentration showed that more than 70% of the cells were densely distributed in the vertical direction (Figure 3C,D). The cell orientation at day 7 was further evaluated using the resulting vector lengths from the images. The results showed that DPSCs on the 400 and 800 nm substrates were highly aligned in one direction, whereas DPSCs on the flat substrate were dispersed in multiple directions (Figure 3E). To determine the proliferation of DPSCs on different substrates, a WST-1 assay was performed. As shown in Figures 3F–G and S5, there were no significant differences between the flat and 800 nm groups on days 1, 4, and 7, regardless of cell seeding concentration. On day 7, cell viability in the 400 nm group was shown to be lower than that in the flat and 800 nm groups (Figure 3G).

**Figure 3:**
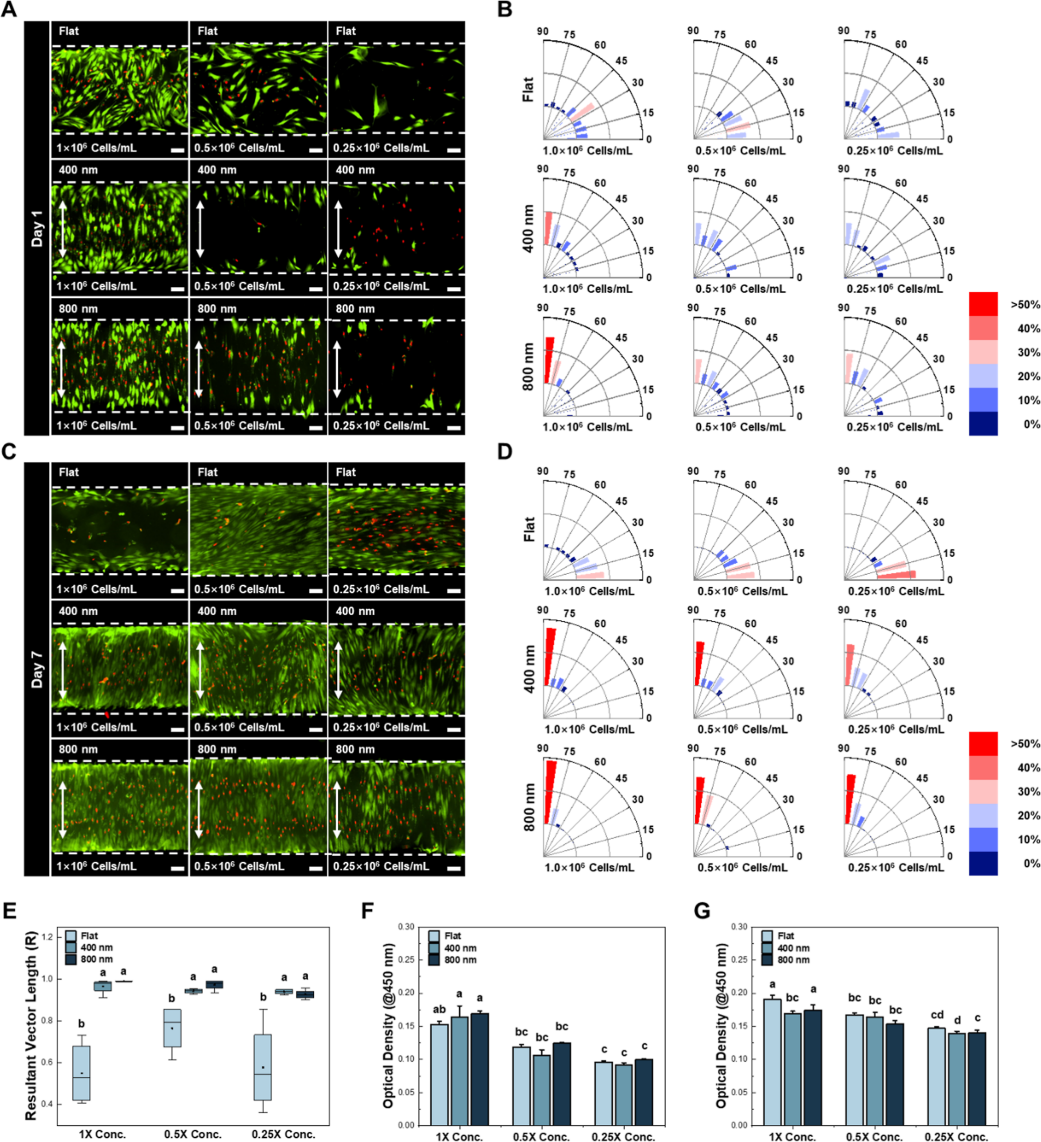
Inducing cell alignment via nanopatterned microchannel with flat, 400 nm, and 800 nm bottom layers. (A) Live/dead images of DPSCs seeded into the microchannel at day 1 (Scale bar 50 μm). (B) The angular frequency distribution of DPSCs corresponding to the live/dead images at day 1. Color maps visually represent the frequency. (C) Live/dead images of DPSCs seeded into the microchannel at day 7. (D) The angular frequency distribution of DPSCs corresponding to the live/dead images at day 7 (Scale bar 50 μm). Color maps visually represent the frequency. The DPSCs were aligned in the direction of the nanopattern, which was perpendicular to the direction of flow. (E) Quantitative analysis of the angular frequency distribution. Box and whisker plots showing the resultant vector length of DPSCs on day 7. The result revealed that nanopatterned substrata significantly enhanced cell alignment. (F) Cell viability of DPSCs seeded into microchannel with flat, 400 nm, and 800 nm bottom layers. WST-1 assay was performed on day 1. (G) Cell viability of DPSCs seeded into microchannel devices with flat, 400 nm, and 800 nm bottom layers. WST-1 assay was performed on day 7. There was no significant differences between flat and 800 nm nanopattern, whereas 400 nm nanopattern showed decreased cell viability (ANOVA, Duncan’s multiple range test, *p* < 0.05). Error bars in (F,G) mean standard errors. WST-1, water-soluble tetrazolium-1.

### 3.4 PDL Regeneration on Bioimplants

To verify PDL formation, periostin expression and collagen formation were evaluated in representative dental stem cell types—DPSCs and PDLSCs. DPSCs cultured on the nanopatterns showed increased periostin expression. In addition, both DPSCs and PDLSCs on the nanopatterns showed different responses depending on the nanopattern. Periostin expression in DPSCs was higher when cultured on 800 nm nanopatterns than on 400 nm nanopatterns. Conversely, periostin expression in PDLSCs was higher when grown on 400 nm nanopatterns than on 800 nm nanopatterns (Figure 4A,B). In addition, Picro-Sirius Red staining was performed to confirm the formation of collagen matrices. The DPSCs and PDLSCs were cultured on the microchannel devices for 7 days. Results indicate that DPSCs cultured on the 800 nm nanopattern showed enhanced collagen formation with highly aligned orientation (Figure 4C,D). DPSCs cultured on microchannels showed better periostin and collagen expression than PDLSCs.

**Figure 4:**
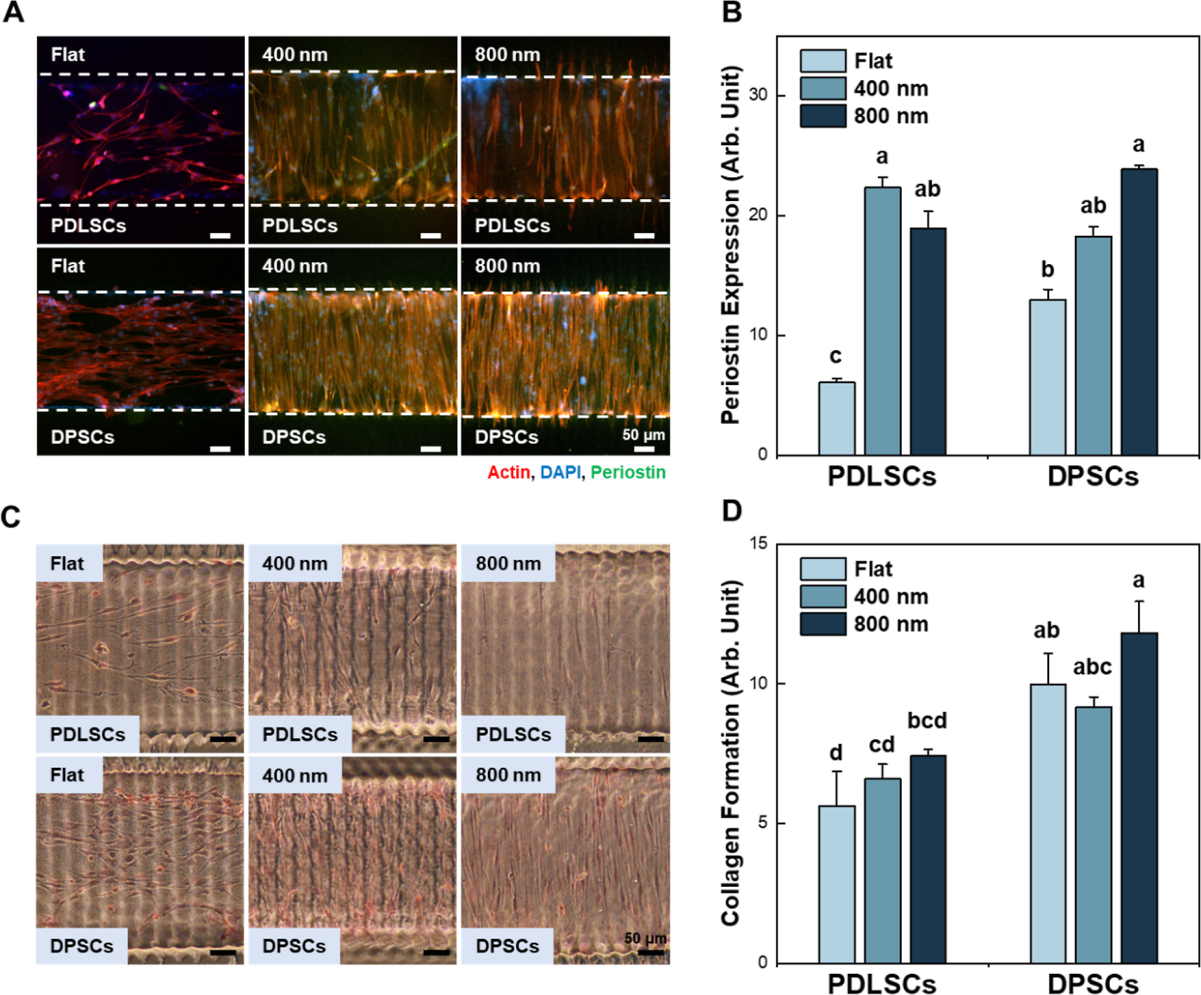
Verifying PDL-like tissue formation in microchannel devices with flat, 400 nm, and 800 nm bottom layers. (A) Immunofluorescence images showing periostin expression on PDLSCs and DPSCs on day 7. (B) Quantitative results of periostin expression corresponding to immunofluorescence images. The expression of periostin in DPSCs was higher when grown with an 800 nm nanopattern than with a 400 nm nanopattern. (C) Microscopic images of the cells stained with Picro-Sirius Red which indicates collagen matrix formation. PDLSCs and DPSCs were seeded into microfluidic devices and cultured for 7 days. The stained region represents collagen matrix. (D) Quantitative results of collagen matrix formation corresponding to Picro-Sirius Red staining. DPSCs cultured on the 800 nm nanopattern exhibited collagenous tissue formation with a highly aligned orientation (ANOVA, Duncan’s multiple range test, *p* < 0.05). Error bars in (B,D) mean standard errors. PDL, periodontal ligament; PDLSCs, periodontal ligament stem cells.

To evaluate PDL regeneration on the bioimplants, bioimplants with different bHA contents were used as follows: pristine Ti (Ti), Ti with 0.05% bHA content (Ti/bHA 0.05), and Ti with 0.5% bHA content (Ti/bHA 0.5). Calcium phosphate cements were incorporated on the opposite side of the bioimplant to mimic the alveolar bone of the implanted site. To evaluate the integration of PDL-like tissue onto the bioimplants, the immunofluorescence images of PDL-like tissue were observed. The attached area of PDL-like tissue on the microchannel was quantitatively analyzed. On day 4, more than 70% of PDL-like tissues adhered to BoC with Ti/bHA 0.05 and Ti/bHA 0.5, whereas less than 30% of PDL-like tissues adhered to BoC with Ti. The expression of cementum protein-1 (CEMP-1) in PDL-like tissues was significantly increased in Ti/bHA 0.05 compared to that of other groups (Figure 5A–C). Furthermore, the spatial distribution of CEMP-1 expression depending on the location of BoC on day 7 is shown in Figure 5D,E. CEMP-1 was expressed at low levels in the CPC region, but was highly expressed in the bioimplant region. These results indicate that PDL-like tissues were successfully attached to the bioimplant and formed a cementum-like structure.

**Figure 5:**
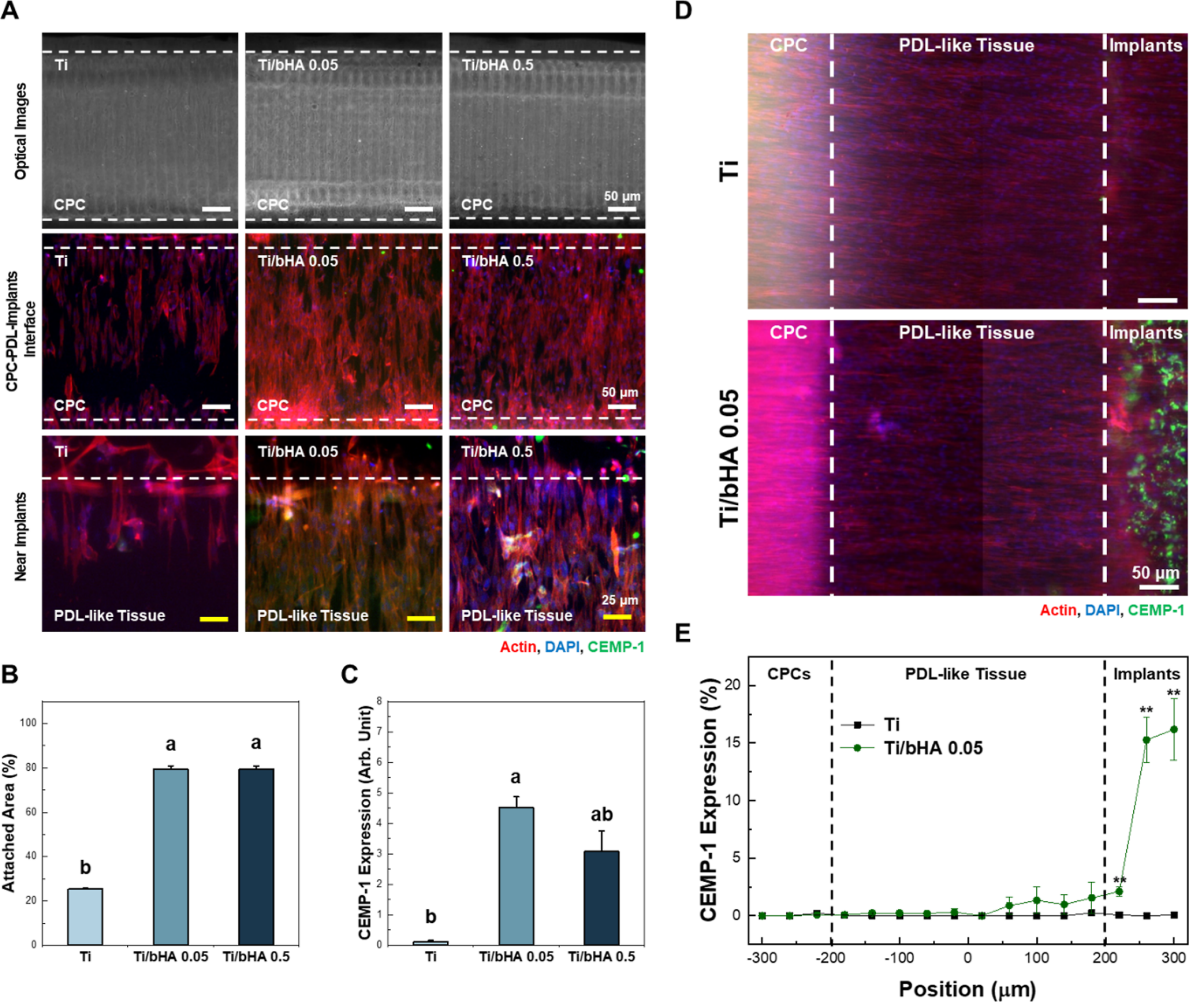
Investigating regeneration of PDL-like tissues on the bioimplants (A) immunofluorescence images of DPSCs cultured in BoC on day 4. Bioimplants with different natural hydroxyapatite contents were incorporated into BoC. Expression of CEMP-1 was observed on Ti, Ti/bHA 0.05 and Ti/bHA 0.5. (B) Quantitative data on attached areas of the PDL-like tissues. The attached area of the PDL-like tissues on Ti/bHA 0.05 showed more than 70%, whereas less than 30% on the BoC with Ti. (C) Quantitative data of CEMP-1 expression corresponding to immunofluorescence images. Expression of CEMP-1 in PDL-like tissues significantly increased in Ti/bHA 0.05 and Ti/bHA 0.5, compared to Ti (ANOVA, Duncan’s multiple range test, *p* < 0.05). Error bars in (B,C) mean standard errors. (D) Microscopic and immunofluorescence images of DPSCs cultured in the BoC with Ti and Ti/bHA 0.05. CEMP-1 expression on day 7 was observed. (E) Spatial distribution of CEMP-1 expression depending on the location. In Ti/bHA 0.05, CEMP-1 was expressed at low levels in the CPC region, but was highly expressed in the bioimplant region. Error bars in (E) mean standard errors (Student’s *t*-test, *p < 0.05 and **p < 0.01). PDL, periodontal ligament; CEMP-1, cementum protein-1; Ti, Ti_6_Al_4_ V; bHA, biogenic hydroxyapatite from equine bone.

## 4 Discussion

Current in vitro periodontal models have several limitations due to their inaccuracy in reproducing the complex microenvironment of periodontium.^17^ They can accurately mimic the microenvironment and physiological characteristics of target tissues or organs; experiments are highly reproducible and controllable.^5^ In addition, organs-on-chips are unencumbered by ethical issues and are cost- and time-efficient, offering several advantages over conventional in vitro or in vivo experiments.^1^ For a more reliable study of PDL regeneration, it is important to develop accurately reproduced periodontal models. In addition, the organ-on-a-chip used to study PDL regeneration can be applied to bioimplant studies. The ultimate goal of implant-based therapy is to replace missing teeth with highly osseointegrative dental implants together with reconstructed periodontium. The goal is to restore masticatory function and the cushioning and preventive effects of the natural PDL. However, extensive studies of dental implants have been conducted only to address the need for improved function and stability in the physiologic environment.^57^ They have generally focused on promoting osseointegration by integrating various surface modification techniques, including sandblasting and acid etching;^58^ calcium phosphate coating;^59,60^ surface functionalization;^61^ and nano/micro patterning.^62,63^ However, conventional dental implants lack the natural periodontium structure, resulting in clinical failure in the long term. Therefore, recent studies have attempted to regenerate PDLs by transplanting dental implants coupled with single or multiple layers of cell sheets.^21,27,64^ The transplanted cell sheets formed not only highly aligned PDL but also the cementum-like tissues on the implant surface, demonstrating the potential to reconstruct the cementum-PDL structure.^28^ Therefore, the development of advanced therapeutic strategies that can reconstruct the PDL-cementum interface around dental implants is of great interest. To date, most studies on PDL regeneration around dental implants have relied on in vivo experiments to confirm PDL-like tissue formation. To overcome the above limitations, a BoC platform that recapitulates the structural features of the periodontium has been developed. This platform allows for easy evaluation of PDL regeneration on the bioimplants in a clinically relevant setting.

The BoC was fabricated using a DLP-based 3D printing technique, which has several advantages over other conventional fabrication techniques. 3D printed microfluidic devices allow multiple materials to be incorporated into the device with a relatively short fabrication time. In addition, complex 3D structures can be incorporated into a microfluidic device.^65^ In particular, DLP-based 3D printing has been used to fabricate molds for microfluidic devices using photocurable resins. DLP-based 3D printing creates a structure by illuminating an entire layer at once, which reduces printing time. In addition, DLP printing allows for easy modification of the design to meet experimental requirements, providing a rapid development process.^66^ Currently, microchannels larger than 100 μm can be fabricated using DLP printing,^65^ which is suitable for mimicking the human PDL.^53^ In this study, the engineered construct was designed with a thickness of 400 μm to reflect the physiological dimensions of the native PDL. To efficiently optimize cell culture conditions in microchannels, a microchannel device with channels identical to those in the BoC was fabricated.^67^ Since DPSCs are grown on nanopatterned PDMS, cell adhesion on PDMS was compared with that on glass, which is commonly used for microfluidic devices (Figure 2A–C). Although there was no significant difference in cell density and cell viability, the DPSCs adhered to PDMS exhibited aggregated or detached behavior due to the hydrophobic nature of PDMS. It has been reported that the surface properties of PDMS can be easily modified by polymer/peptide or ECM coatings.^68^ Cells often show differential proliferation and adhesion depending on the ECM.^69^ Three different ECM matrices suitable for surface coating of microchannels were tested and it was confirmed that fibronectin showed the highest cell adhesion among the ECM materials (Figure 2D,E). The conditions for fibronectin coating were further investigated at different time and temperature points. The optimal conditions were confirmed to be overnight incubation at room temperature (Figure S3). DPSCs under optimal conditions showed improved attachment regardless of cell concentration, whereas DPSCs in other groups did not adhere properly throughout the channel. Fibronectin is a major cell adhesion protein that contains an arginine-glycine-aspartate (RGD) sequence.^70^ The RGD motif in fibronectin has been suggested to enhance the attachment and spreading of DPSCs by acting as an integrin-binding signaling molecule.^71^

To replicate the highly aligned architecture of native PDL tissue, nanotopographic cues were introduced in order to induce cellular alignment. Nanogrooved PDMS substrates with feature sizes of 400 and 800 nm were selected based on previous studies demonstrating that groove dimensions in this range effectively induce alignment, elongation, and extracellular matrix organization in various soft tissue models, including tendon,^72^ muscle^73^ and neural regeneration.^74^ These nanoscale features provide critical biophysical cues that mimic the anisotropic organization of native extracellular matrices and modulate key cellular behaviors involved in tissue integration. Using nanopatterned PDMS, highly oriented cell alignment perpendicular to the flow was successfully induced in the devices (Figure 3A–D). The nanopattern with 800 nm grooves provided more suitable guidance to induce cell alignment compared to the flat and 400 nm groups on day 7, without interfering with cell proliferation (Figure 3E–G). The anisotropic physical properties of the ECM can provide guidance cues and may play a critical role in modulating cell and tissue functions.^75^ The nanopatterned substrate induced cell alignment, which may have contributed to the subsequent PDL-like tissue formation.^76^ Comparative studies of collagen formation and periostin expression provided further evidence for ligamentous soft tissue formation. The expression of periostin, a matri-cellular PDL-specific marker found in collagen-rich connective tissue, confirmed that the DPSCs could form PDL-like tissue.^76^ Furthermore, the formation of densely aligned collagen fibers in DPSCs was even higher than that of PDLSCs (Figure 4). Finally, the integration of PDL-like tissues onto bioimplants with different bHA contents, Ti, Ti/bHA 0.05, and Ti/bHA 0.5, was evaluated using BoC. The PDL-like tissues anchored on Ti/bHA 0.05 showed significantly higher expression of CEMP-1. In particular, spatial distribution of CEMP-1 expression verified that the bioimplants incorporated with bHA had a greater ability to form a cementum-like structure compared to Ti, successfully reproducing the cementum-PDL-alveolar bone interface. Cementum plays an important role in regulating local metabolism and differentiation in PDL. The formation of the cementum-like structure is attributed to bHA, which has excellent biocompatibility, osteogenic capacity, protein adsorption properties, and chemical similarities to bone mineral.^77,78^ In addition, HA has been reported to promote cementogenic differentiation of dental stem cells, which may have contributed to the formation of cementum-like tissue.^79^

In this study, a novel BoC is proposed for the evaluation of PDL-like tissue formation on bioimplants. The proposed platform is the first to incorporate a dental implant into a microfluidic device and construct a dental implant-PDL interface. This has several implications for dental implant therapy. First, BoCs have implant materials embedded in the device and can be exchanged easily. Therefore, it is possible to study the biological response to dental implants as well as different biomaterials. Second, because the BoCs were able to successfully mimic the cementum-PDL interface, indicative of resemblance to natural periodontal tissues, they are able to provide more reliable results compared to that of other conventional in vitro tests or models. Third, BoCs have potential applications in personalized medicine. Fabrication of BoCs using patient-derived stem cells in BoCs would provide an accurate model of personalized responses to dental implants. In addition, the platform serves as a powerful in vitro evaluation system for next-generation implant designs. Its modular architecture enables systematic testing of novel implant concepts under controlled conditions. Furthermore, the system can be adapted as a disease model, expanding its utility beyond baseline tissue integration studies. For instance, by incorporating microbial components such as oral pathogens, the platform could simulate peri-implantitis, a leading cause of implant failure characterized by inflammation and soft tissue degradation. This adaptation would allow for real-time assessment of tissue barrier integrity, host–microbe interactions, and potential antimicrobial strategies. Furthermore, the platform may be adapted to recapitulate hypoxic microenvironments that typically occur after surgical implantation due to temporary disruption of local vasculature.^80^ With the thickness of the microchannels being set to 400 μm, the current system was able to maintain normoxic conditions by passive oxygen diffusion alone. We recognize that future versions could integrate oxygen-controlling elements, such as low-permeability channel materials, perfusion-free regions, or chemical hypoxia inducers, to establish oxygen gradients or simulate transient hypoxia.^81^ This would enhance the relevance of the model in evaluating early stage implant performance under conditions that better reflect the regenerative challenges encountered in vivo. Finally, while perpendicular flow was intentionally avoided in the current model to preserve topography-guided cellular alignment during the early stages of PDL-like tissue formation, the introduction of such flow in future versions of the platform would enable the simulation of physiologically relevant conditions. In vivo, oral fluids such as saliva and crevicular fluid naturally exert shear forces perpendicular to the alignment of periodontal tissues,^82,83^ and mastication generates lateral mechanical stress across the implant–tissue interface.^84^ Incorporating controlled perpendicular flow would allow for the investigation of how these dynamic mechanical cues influence tissue remodeling, cytoskeletal adaptation, and mechanotransductive signaling, thereby increasing the model’s fidelity to the in vivo postimplantation environment.

Despite these strengths and opportunities for expansion, several limitations of the current platform should be acknowledged. The model does not yet replicate the full complexity of the in vivo environment, including systemic immune interactions, long-term mechanical loading, or dynamic remodeling over extended culture periods. Additionally, the scalability of the system for high-throughput testing and the long-term maintenance of tissue function under static conditions remain technical challenges. Addressing these limitations in future iterations—such as by incorporating immune cells, perfusion systems, or modular mechanical actuators—will be essential to further enhance the platform’s translational relevance. Together, these future enhancements and critical considerations will not only improve the clinical applicability of the model but also broaden its utility in research on implantology, soft tissue integration, and personalized regenerative strategies.

## 5 Conclusions

In this study, we developed a novel BoC platform that successfully recapitulates the structural and functional features of the native periodontium for in vitro evaluation of dental implants. The platform integrated a titanium-based bioimplant with biogenic hydroxyapatite, calcium phosphate cement, and a nanogrooved PDMS substrate to support the formation of aligned PDL-like tissue using human dental pulp stem cells. By reproducing the cementum–PDL interface and inducing ligament-specific marker expression, the BoC enabled the evaluation of early soft tissue integration and cementogenesis in a controlled, physiologically relevant microenvironment. Systematic optimization of cell seeding, extracellular matrix coatings, and topographic guidance demonstrated the platform’s ability to mimic native alignment and support collagenous matrix formation. Moreover, the platform revealed material-dependent differences in CEMP-1 expression and tissue attachment, highlighting its utility for comparative implant screening. While designed for static, normoxic conditions to preserve alignment, the modular architecture of the BoC supports future incorporation of mechanical, microbial, and hypoxic stimuli to further increase physiological fidelity. Altogether, this work provides a robust, scalable, and translationally relevant in vitro model for probing implant–tissue interactions, with potential applications in bioimplant development, regenerative dentistry, and personalized therapeutic screening.

## Supplementary Material



## Notes

Optimization of cell seeding density on microchannel devices, qualitative analysis of cellular adhesion, optimization of fibronectin coating, analysis of cellular alignment, and cell viability assay on different microchannel devices (PDF)
