# Supplementary material for: Bioimplant-on-a-Chip for Facile Investigation of Periodontal Ligament Formation on Biogenic Hydroxyapatite/Ti6Al4 V Implants
Source: ACS Appl Mater Interfaces. 2025 May 13;17(21):30673–85. doi: 10.1021/acsami.5c04687 (PMC12123576; doi:10.1021/acsami.5c04687)
Supplement: Supplementary file 1 [file am5c04687_si_001.pdf]

Supporting information

Bioimplant-on-a-Chip for Facile Investigation of Periodontal Ligament Formation on Biogenic Hydroxyapatite/Ti6Al4V Implants

Sangbae Park[1,2,3,†], Jae Eun Kim[1,†], Juo Lee[4], Woochan Kim[5,6,7], Woobin Choi[1], Myung Chul Lee[8], Jae Woon Lim[1], Kyoung-Je Jang[9,10,*], Hoon Seonwoo[4,11,*], Jangho Kim[5,6,7,*], Jong Hoon Chung[1,*]

[1] Department of Biosystems Engineering, Seoul National University, Seoul 08826, Korea

[2] Research Institute of Agriculture and Life Sciences, Seoul National University, Seoul 08826, Republic of Korea

[3] Integrated Major in Global Smart Farm, College of Agriculture and Life Sciences, Seoul National University, Seoul 08826, Republic of Korea

[4] Department of Convergent Biosystems Engineering, College of Life Science and Natural Resources, Sunchon National University, Suncheon, 57922, Republic of Korea

[5] Department of Convergence Biosystems Engineering, Chonnam National University, Gwangju 61186, Republic of Korea

[6] Department of Rural and Biosystems Engineering, Chonnam National University, Gwangju 61186, Republic of Korea

[7] Interdisciplinary Program in IT-Bio Convergence System, Chonnam National University, Gwangju 61186, Republic of Korea

[8] Medicinal Materials Research Center, Biomedical Research Division, Korea Institute of Science and Technology (KIST), Seoul, 02792 Republic of Korea

[9] Department of Bio-Systems Engineering, Institute of Smart Farm, Gyeongsang National University, Jinju 52828, Republic of Korea

[10] Institute of Agriculture & Life Science, Gyeongsang National University, Jinju 52828, Republic of Korea

[11] Interdisciplinary Program in IT-Bio Convergence System, Sunchon National University, Suncheon, 57922, Republic of Korea

†These authors equally contributed to this work

*Correspondence: Jong Hoon Chung (jchung@snu.ac.kr), Jangho Kim (rain2000@jnu.ac.kr), Hoon Seonwoo (uhun906@gmail.com), and Kyoung-Je Jang (kj_jang@gnu.ac.kr)

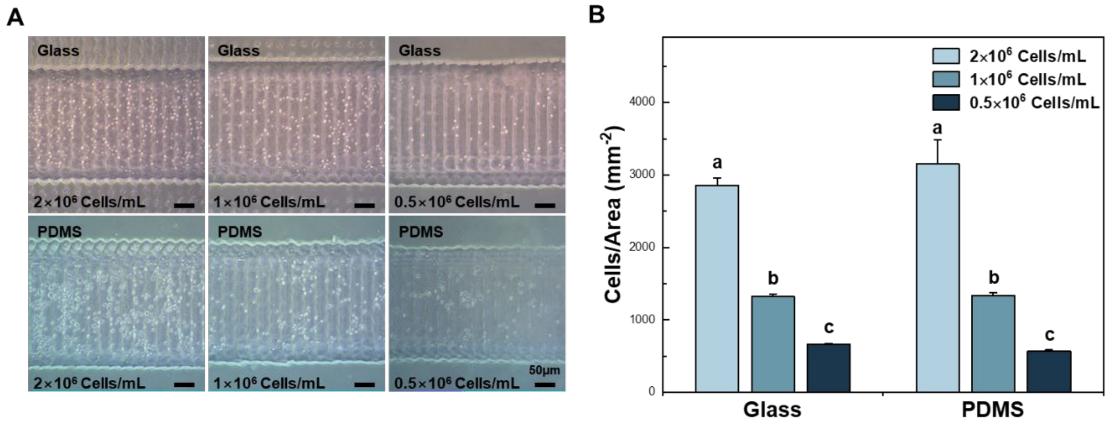

Figure S1. Microchannel cell culture with various seeding concentrations. (A) Microscopic images of seeded DPSCs. DPSCs were well seeded into the microchannels and even distribution was confirmed. (B) Seeding density of DPSCs in the microchannels. (ANOVA, Duncan's multiple range test, $p < 0.05$). Error bars in (B) mean standard errors.

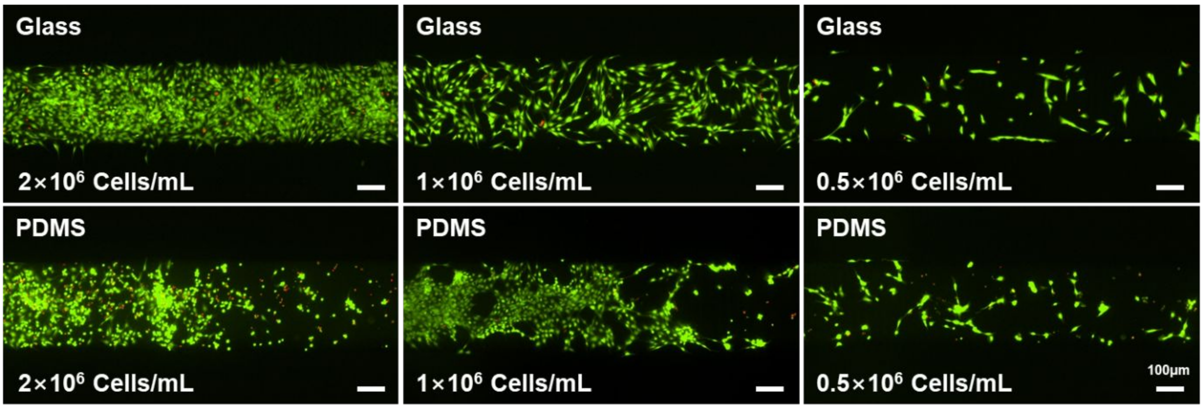

Figure S2. Live/Dead images of DPSCs seeded onto glass- and PDMS-bottomed microchannels (Magnification: 40X). DPSCs on the PDMS showed aggregation or uneven distribution.

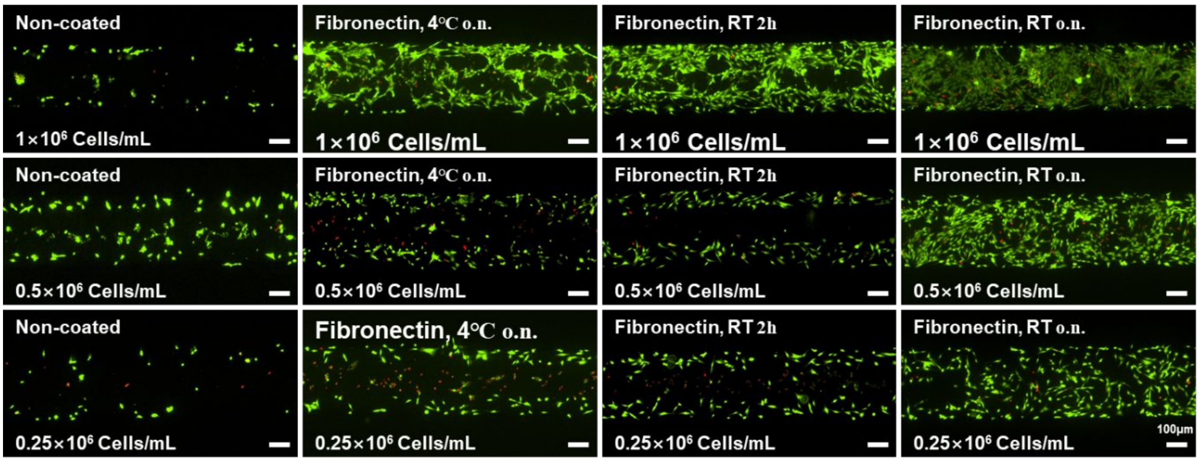

Figure S3. Fibronectin coating of microchannels at different coating conditions. Fibronectin coated at room temperature overnight showed the most enhanced cell adhesion among other groups.

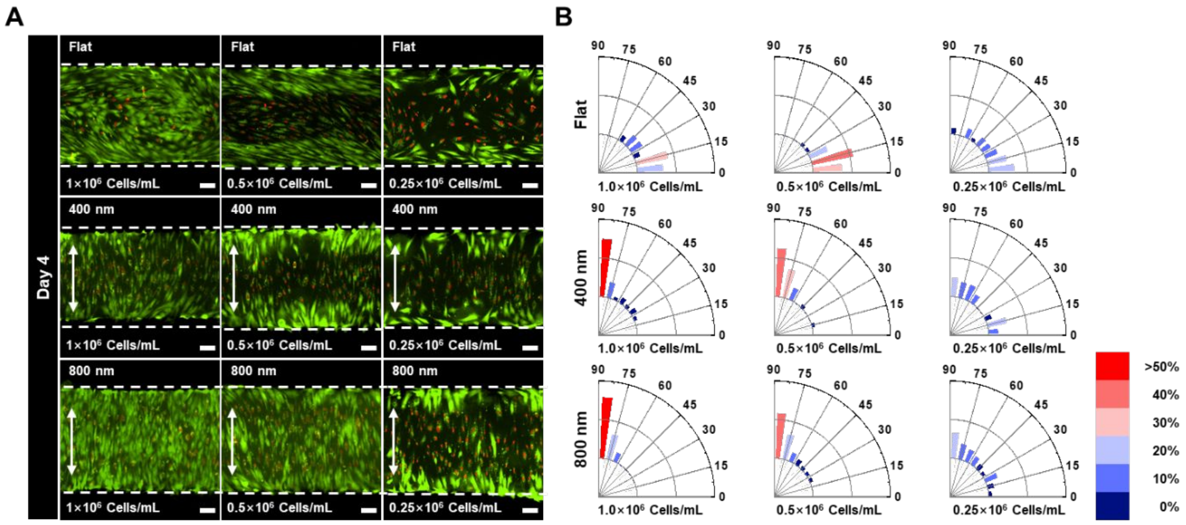

Figure S4. Inducing cell alignment via nanopatterned microchannel with flat, 400 nm, and 800 nm bottom layers. (A) Live/dead images of DPSCs seeded into the microchannel at day 4 (Scale bar 50 μm). (B) The angular frequency distribution of DPSCs corresponding to the live/dead images at day 1. Color maps visually represent the frequency.

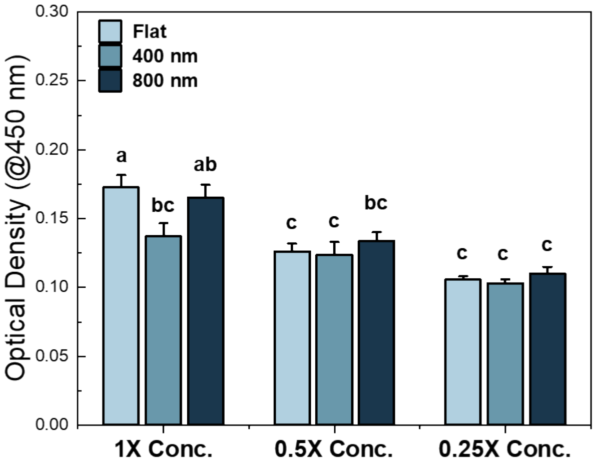

Figure S5. Cell viability of DPSCs seeded into microchannel devices with flat, 400 nm, and 800 nm bottom layers. WST-1 assay was performed on day 4. There were no significant differences between flat and 800 nm nanopattern, whereas 400 nm nanopattern showed decreased cell viability (ANOVA, Duncan's multiple range test, $p < 0.05$). Error bars mean standard errors.
